# Impact of Oropharyngeal Packing on Postoperative Nausea, Vomiting, and Throat Discomfort: A Systematic Review and Meta-Analysis

**DOI:** 10.3390/dj14060337

**Published:** 2026-06-02

**Authors:** Roger Alonso-Royo, Rocío Trinidad Velázquez-Cayon, Pilar Martín-Balbuena, Álvaro-José Rodríguez-Domínguez, Carmen María Sánchez-Torrelo

**Affiliations:** 1 Department of Ciencias Sociales y de la Salud, Centro Department of Ciencias Sociales y de la Salud, Universitario San Isidoro, Affiliated with Pablo de Olavide University (UPO), 41092 Sevilla, Spain; rvelazquez@centrosanisidoro.es (R.T.V.-C.); pilarmartinbalbuena@centrosanisidoro.es (P.M.-B.); arodriguezdominguez@centrosanisidoro.es (Á.-J.R.-D.); 2 Department of Physiotherapy, University School of Osuna, University of Seville, 41640 Sevilla, Spain; csanchezt@euosuna.org

**Keywords:** throat pack, PONV, throat discomfort

## Abstract

**Background and Objectives**: The insertion of a throat pack is a routine practice during anesthesia in maxillofacial and upper airway surgeries to prevent the aspiration of blood and secretions. However, this technique is associated with postoperative adverse effects such as pharyngitis and nausea. The objective of this systematic review and meta-analysis was to analyze the clinical evidence regarding the effect of throat packs on the incidence of Postoperative Nausea and Vomiting (PONV), throat pain, and gastric volume. **Materials and Methods**: Conducted according to PRISMA 2020 and registered in PROSPERO (CRD420251027016), an exhaustive search was performed in PubMed, Scopus, WOS, and Cochrane Library. Randomized Controlled Trials (RCTs) comparing throat pack use versus non-use under general anesthesia were included. Quality was assessed using Cochrane RoB 2. Meta-analyses utilized Standardized Mean Difference (SMD) and Mean Difference (MD) through a random-effects model. **Results**: Fourteen articles (2002–2024) involving 1147 patients were included. Methodological quality predominantly raised “some concerns”, with only 7.1% of studies assessed at low risk of bias. Statistical analysis of pain intensity (k = 6) revealed a significant increase in the intervention group (SMD = 0.35; 95% CI: 0.09 to 0.62; *p* = 0.01). Regarding PONV (k = 3), no statistically significant differences were observed between groups (SMD = −0.47; 95% CI: −1.51 to 0.58; *p* = 0.38). Ultrasound measurements showed significant increases in gastric parameters in the throat pack group, including anteroposterior diameter (MD = −3.67 mm; *p* = 0.002) and antral cross-sectional area (MD = −223.10 mm^2^; *p* = 0.005). **Conclusions**: Current evidence does not demonstrate a clear benefit for the routine use of oropharyngeal packing in maxillofacial and upper airway surgeries, and suggests a possible increase in postoperative throat discomfort. While acknowledging the limited number of studies, clinical heterogeneity, and the influence of perioperative co-interventions, these results should be interpreted with caution.

## 1. Introduction

The use of a throat pack is widely described in maxillofacial and upper airway surgeries to secure the airway with a tracheal tube (or, sometimes, with a supraglottic device). The purpose of this routine practice is to absorb any residue of blood, secretions, or other fluids that may accumulate during the procedure. By keeping the airway clean, the formation of clots that, upon detachment after surgery (known as a “forensic clot”), could dangerously obstruct the patient’s airways is prevented [1].

A throat pack is generally inserted for six primary reasons: to prevent air leaks (more common in children), to prevent blood or other substances from contaminating the lungs, to absorb nasal fluids that might drip into the throat, to facilitate surgeries in the area, to secure the airway when a flexible laryngeal mask airway is used in nasal surgeries, and to prevent blood from reaching the stomach, which could cause postoperative discomfort [1].

Throat packing is associated with problems such as pharyngitis or sore throat, which is a discomfort located in and around the pharynx with a diverse etiology [2], nausea, which is the unpleasant sensation of needing to vomit. Increased salivation may accompany nausea, and vomiting is the forced expulsion of stomach contents through the mouth [3]. Nausea and vomiting are a very common condition in the postoperative period of maxillofacial and upper airway surgeries [4] and have been thoroughly investigated to determine the factors that cause them [5].

The objective of this investigation is to analyze the scientific evidence regarding the effect of throat pack placement during maxillofacial and upper airway surgeries.

## 2. Materials and Methods

The PRISMA (Preferred Reporting Items for Systematic Reviews and Meta-Analyses) guidelines were adhered to in guiding this review [6]. The study protocol was registered in the PROSPERO database with the identification number CRD420251027016.

### 2.1. Focused Question

In patients undergoing maxillofacial and upper airway surgeries, does the use of a throat pack prevent Postoperative Nausea and Vomiting (PONV), affect gastric content, or cause postoperative throat discomfort?

The PICO strategy was employed to frame the eligibility criteria. Included studies needed to address all components of PICO strategy, as detailed below:

- Participants: adults who have undergone maxillofacial and upper airway surgeries.
- Intervention: throat pack.
- Comparison: no throat pack
- Outcomes: PONV (Postoperative Nausea and Vomiting), postoperative throat discomfort, and gastric volume.
- Studies: Randomized clinical trial.

### 2.2. Information Sources and Search Strategy

The bibliographic search, conducted in September 2025, encompassed databases such as PubMed, Scopus, Web of Science (WOS) and Cochrane Library. Employing Medical Subject Headings (MeSHs) (“Surgical Sponges”, “tampons, surgical”, “surgery oral”, “Oral Surgical Procedures”, “Orthognathic Surgery”, “Mandibular Reconstruction”, “Anesthesia”, “anesthesia, general” and “anesthesia, endotracheal”) in combination with Boolean operators “AND” and “OR,” a tailored search strategy was made (see Table S1 in Supplementary Material). A search for additional articles was also conducted by tracking the bibliographies of related studies.

### 2.3. Eligibility Criteria

Specific inclusion criteria were applied to select the pertinent studies. The articles had to be Randomized Controlled Trials (RCTs) that compared the application of a throat pack versus non-application during surgery under general anesthesia. Furthermore, the intervention in the throat pack group had to be limited to a dry pack or one dampened with water or saline solution, excluding other agents such as anesthetics or iodine. Crucially, eligible studies were required to include subjects evaluated for Postoperative Nausea and/or Vomiting (PONV), postoperative throat discomfort as part of their clinical assessment or gastric volume as part of their clinical assessment.

Conversely, low-quality studies or those that were not available in full text were excluded.

### 2.4. Selection Process

Following comprehensive database searches, duplicate records were systematically removed using the Zotero reference management software (version 6.0.26). The initial screening phase entailed an evaluation of titles and abstracts, followed by a full-text review to determine final eligibility. Inclusion and exclusion criteria were rigorously and consistently applied throughout the selection process. Two independent reviewers (R.A.-R. and R.T.V.-C.) conducted the preliminary screening, while a third reviewer (C.M.S.-T.) adjudicated any discrepancies. The inter-rater reliability in the screening phase was evaluated using Cohen’s Kappa coefficient (kappa), calculated on a total of 1858 records independently evaluated by two reviewers. The analysis yielded a coefficient of 0.799 (*p* < 0.001), representing a substantial agreement according to Landis and Koch’s criteria and demonstrating the methodological robustness of the selection process. The absolute agreement was 99.78%, with unanimous coincidence in 1840 exclusions and 8 inclusions. Additionally, 6 more articles found manually by tracking the bibliographies of related studies were included. The 4 identified discrepancies (0.22% of the total) were resolved through mutual consensus after reviewing the eligibility criteria, thus ensuring the transparency and reproducibility of the final sample.

### 2.5. Data Collection Processing and Data Items

Key information from the included studies was collected, such as study characteristics, intervention details, and outcomes. Discrepancies in the extracted data were resolved through discussion among the reviewers or, if necessary, by consulting a third reviewer. In some cases, the original authors were contacted to obtain clarifications or additional data.

The evaluation of throat pain in the different articles of this systematic review was addressed through a diversity of instruments that combine quantitative and qualitative approaches, reflecting the heterogeneity of the included studies. Quantitative measurements of pain intensity were mainly carried out using the “Sore throat intensity scale” (with a range of 1 to 6) and the Visual Analog Scale (VAS), which measures pain from 0 to 10. On the other hand, qualitative or categorical assessment methods were more varied, including: the categorized form of the VAS (with levels of “None, mild, moderate, and severe”), a similar Likert scale with the same categories (“none, mild, moderate, and severe”), a scale that compares the pain to a cold, categorizing it as 0: None, 1: Less severe than a cold, 2: Similar to a cold, and 3: More severe than a cold, a dichotomous “Yes or No” response, and the Wong–Baker FACES Pain Rating Scale. This variety of scales and methodologies was used by the authors of the primary studies to measure this outcome.

For this review, data were specifically sought for the presence of symptoms of throat pain (measured using the VAS or NPRS) and the presence of Postoperative Nausea and Vomiting (PONV) (assessed using the Kortilla scale); it is specifically defined as any nausea, retching, or vomiting occurring immediately after the operation and at 24 h following a maxillofacial and upper airway surgeries, and gastric volume. All outcomes that adhered to these endpoints were included, regardless of the measurement scale used or the moment at which follow-up was performed. In studies with multiple temporal assessments, the corresponding data for each were collected. The selection of the outcomes to be extracted was based on their clinical relevance and their coherence with the objectives of this review.

Tables S2 and S3 summarize the main characteristics of the studies using dry and wet throat packs, respectively. Participant demographics and clinical baseline data are detailed in Table S4. Detailed intraoperative data and long-term postoperative outcomes are available in Tables S5 and S6 of the Supplementary Material.

Unit-of-analysis errors were prevented by categorizing outcomes into distinct time-frames (e.g., immediate, short-term, and long-term). For each meta-analysis, only a single observation per study was used; in cases where multiple measurements were reported, the one closest to 2 h post-intervention was selected. In those studies with more than two intervention arms, several comparisons were performed in the meta-analysis by splitting the sample in the duplicated group to avoid overestimating the results. To avoid double counting participants, we appropriately split the control group sample size when the studies compared two different ET modalities [7,8].

### 2.6. Risk of Bias Assessment

The methodological quality of the studies was assessed using the Cochrane Risk of Bias tool for randomized trials (RoB 2). Each study was independently evaluated, and the overall risk of bias was categorized as “low risk,” “some concerns,” or “high risk.” Two authors independently examined each study, assessing the specific domains required by the tool: bias arising from the randomization process, bias due to deviations from intended interventions, bias due to missing outcome data, bias in measurement of the outcome, and bias in selection of the reported result. Any discrepancy that arose during the evaluation was resolved through discussion between the reviewers or, if consensus was not reached, with the intervention of a third author, thus ensuring an exhaustive and consensual assessment of the methodological quality of the included studies (Figure S2).

### 2.7. Effect Measures

The objective of this systematic review was to evaluate the efficacy of the throat pack, designating Postoperative Nausea and Vomiting (PONV) as the primary outcome. PONV severity was assessed using Korttila’s scale, a descriptive verbal scale ranging from 0 (no nausea or vomiting) to 4 (more than one vomiting episode). As another primary outcome, postoperative throat pain was evaluated using either the Visual Analog Scale (VAS) or the Numeric Pain Rating Scale (NPRS), where pain intensity is scored from 0 (no pain) to 10 (the worst imaginable pain).

Other secondary variables included gastric volume assessment via ultrasound, recording the anteroposterior and cranio-caudal diameters (mm), the antral cross-sectional area (mm^2^), and the total gastric volume (ml); these measurements were taken both preoperatively and postoperatively to evaluate the Mean Difference. Additionally, surgical and anesthesia durations were recorded in minutes, and total blood loss was measured in ml. The presence of bloody gastric content was also monitored. Furthermore, swallowing difficulty was assessed postoperatively using a specific swallowing difficulty scale. Finally, throat inflammation was evaluated after the procedure using a 6-point Likert scale, ranging from 1 (total absence of inflammation) to 6.

### 2.8. Transformation of Variables

To ensure clinical and statistical comparability across studies, data were harmonized using a principled approach. For continuous outcomes such as pain scores, the Standardized Mean Difference (SMD) was utilized to account for the diversity of measurement scales across trials. Regarding PONV and throat discomfort, although some studies reported ordinal severity scales, these were systematically dichotomized into binary incidence outcomes (presence vs. absence of symptoms). This pre-planned transformation was conducted to allow for a robust pooled analysis of overall incidence, considering any degree of affectation (from mild to severe) as a positive event. We believe this approach minimizes clinical heterogeneity and provides a more conservative and transparent estimate of the treatment effect.

### 2.9. Synthesis Methods

A quantitative synthesis was performed to evaluate the clinical efficacy of throat packs compared to no pack intervention. The primary outcomes analyzed were pain intensity, Postoperative Nausea and Vomiting (PONV) and ultrasound measurement. Data extraction focused on means and standard deviations (SDs) to facilitate the meta-analysis.

In cases where studies utilized different assessment tools or scales for the same outcome (such as pain intensity or PONV), the Standardized Mean Difference (SMD) and its corresponding Standard Error (SE) were calculated to ensure comparability. Conversely, for ultrasound variables where all studies reported measurements in the same unit (millimeters), the Mean Difference (MD) was utilized. In cases where multiple measurements were reported, the one closest to 2 h post-intervention was selected.

To account for clinical and methodological diversity, subgroup analyses were conducted based on the type of throat pack used: wet, dry, or not specified. A random-effects inverse-variance meta-analysis using the DerSimonian–Laird estimator for between-study variance (τ^2^) was conducted, as implemented in Review Manager 5.4, given the expected clinical and methodological heterogeneity across studies. Therefore, no I^2^ cutoff was used to determine the choice of model. Prediction intervals were calculated for each meta-analysis using a random-effects model, incorporating between-study variance (τ^2^) to estimate the range in which the true effect of a future study is expected to lie [7].

Publication bias was evaluated for meta-analyses comprising more than 10 studies. Additionally, a sensitivity analysis was conducted to determine the individual influence of each study on the overall pooled effect. All statistical analyses were performed using Review Manager version 5.4.1.

### 2.10. Certainty Assessment

Certain pieces of evidence were assessed using the GRADE framework (Grading of Recommendations, Assessment, Development and Evaluation) [6,7], Outcome-level certainty judgments were established, and evidence was downgraded based on specific factors such as risk of bias, inconsistency, and imprecision, without assuming an automatically high quality for randomized trials. Table 1 details the assessment of the outcomes.

## 3. Results

### 3.1. Results of the Search

**Figure 1:**
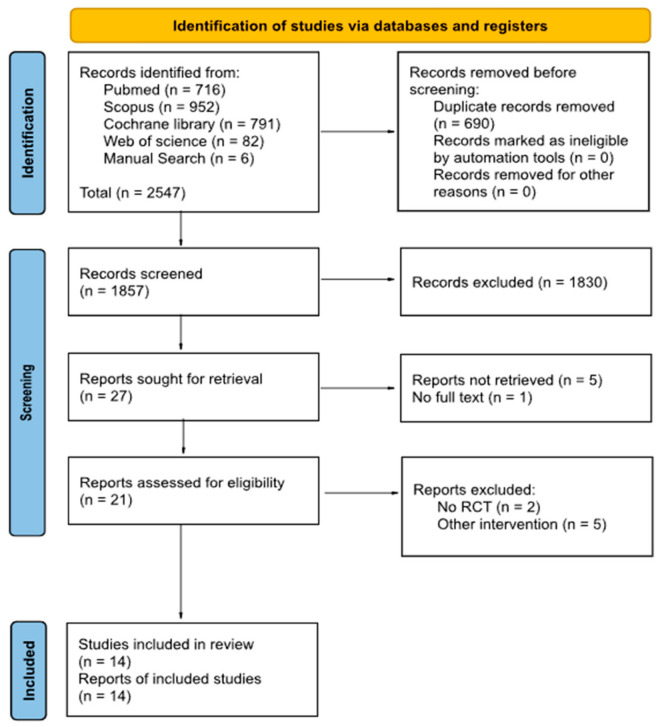
PRISMA flowchart.

The initial database search, conducted without filters, returned 2547 studies: 716 from PubMed, 952 from Scopus, 791 from Cochrane Library, 82 from Web of Science, and 6 articles found manually by tracking the bibliographies of related studies. A total of 689 studies were eliminated due to duplication, leaving 1858 articles, of which 1830 articles were removed after differentiating by title and abstract, leaving 28 articles. Finally, 14 articles were included in the review [9,10,11,12,13,14,15,16,17,18,19,20,21,22] (see Figure 1).

### 3.2. Study Characteristics

This systematic review focused exclusively on Randomized Controlled Trials, totaling 1147 patients distributed in an intervention group of 569 and a control group of 578; the studies spanned from the year 2002 to 2024. Orthognathic surgeries [10,15] were performed, and the rest were nasal surgeries [9,11,14,16,17,18,19,20,21]. The provenance of the included studies in this analysis is varied, with Turkey [13,14,16] and the United States [9,10,12] being the most represented countries, each with three investigations. They were followed by Brazil, with two studies [15,19], and finally Saudi Arabia [11], England [17], Iran [18], Ireland [20], Singapore [21] and Germany [22], each with one study (see Tables S2–S4 in the Supplementary Material for full details). Additionally, four studies placed the throat pack in the oropharynx [9,10,15,17], nine placed it in the pharynx [11,12,13,16,18,19,20,21,22], and one study in the hypopharynx [14].

### 3.3. Risk of Bias

The risk of bias assessment using the RoB 2 tool for the 14 included studies reveals that only 7.1% of the trials present a low overall risk of bias, while 64.3% show “some concerns” and 28.6% are classified as “high risk.” Regarding the specific domains, the randomization process presented “some concerns” in 78.6% of the cases, with 7.1% at high risk and only 14.3% at low risk. In the domain of deviations from intended interventions, 71.4% of the studies showed a low risk, while 14.3% were equally distributed between “some concerns” and “high risk.”

In terms of missing outcome data, 78.6% of the papers achieved a low-risk rating, although a significant 21.4% were categorized as high risk. Measurement of the outcome was the domain with the greatest methodological strength, reaching 85.7% low risk, compared to 7.1% with some concerns and 7.1% at high risk. Finally, in the selection of the reported result, 64.3% of the studies raised some concerns, while the remaining 35.7% were considered low risk, with no high-risk cases detected in this specific domain (see Figure S1 in Supplementary Material).

### 3.4. Results of Individual Articles According to Symptomatology

Regarding the search for articles on PONV, we found 14 articles: Borna et al. [9] in 2022 found no differences in the incidence of Postoperative Nausea and Vomiting (PONV) between the intervention group and the control group. However, they did observe that the use of the pharyngeal pack worsened throat pain; Powel et al. [10] in 2022 reported that the throat pack did not improve PONV, increased postoperative throat discomfort, and was not an effective barrier to prevent the ingestion of blood during the operation; Al-Jandan et al. [11] in 2018 found a higher incidence of PONV in the group without a guide. Regarding throat pain, the study found no differences between the pharyngeal pack groups (guided or unguided) compared to the control group, but the use of the pack did cause greater throat discomfort overall; Green et al. [12] in 2017 found no significant differences in throat discomfort at 4 h, but did find more discomfort in the intervention group at 24 h compared to the control group; Korkut et al. [13] in 2010 reported that there was no difference in the incidence of PONV between the intervention group and the control group; Altun et al. [14] in 2024 reported that the throat pack reduces PONV, decreases throat discomfort compared to the control group, and reduces stomach volume; Faro et al. [15] in 2020 reported that the throat pack reduces PONV, decreases throat discomfort compared to the control group, and reduces stomach volume; Temel et al. [16] in 2019 reported that the use of the throat pack reduces PONV and also stomach volume; Al-lami et al. [17] in 2017 found no differences in the incidence of PONV between the intervention group and the control group. Regarding throat pain, they found greater discomfort in the pack group, but no significant difference at 2 and 6 h; Razavi et al. [18] in 2015 found no difference between the intervention and control groups regarding PONV and throat discomfort; Piltcher et al. [19] in 2007 found no differences in the incidence of PONV between using or not using the throat pack; Basha et al. [20] in 2006 reported that PONV does not decrease due to the use of the pharyngeal pack and that it also causes throat discomfort; Tay et al. [21], in 2002, found no difference in throat discomfort between using or not using the throat pack, and Pabst et al. [22] found increased symptomatology due to the use of the throat pack (see Tables S5 and S6 in Supplementary Materials).

### 3.5. Results of Quantitative Analysis Syntheses

#### 3.5.1. Postoperative Pain

**Figure 2:**
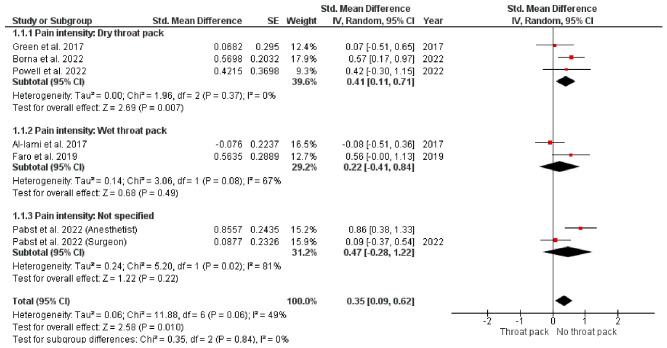
Forest plot for pain intensity: throat pack versus no throat pack [9,10,12,15,17,22].

Six studies were included in the meta-analysis for the pain intensity variable. Statistically significant results were observed favoring the no throat pack group overall (SMD = 0.35; 95% CI [0.09, 0.62; *p* = 0.01]. This trend was specifically maintained in the dry pack subgroup (SMD = 0.41; 95% CI [0.11, 0.71; *p* = 0.007]. The prediction interval ranged from −0.27 to 0.97. Figure 2 shows the forest plot for the pain intensity variable.

#### 3.5.2. Postoperative Nausea and Vomiting

**Figure 3:**
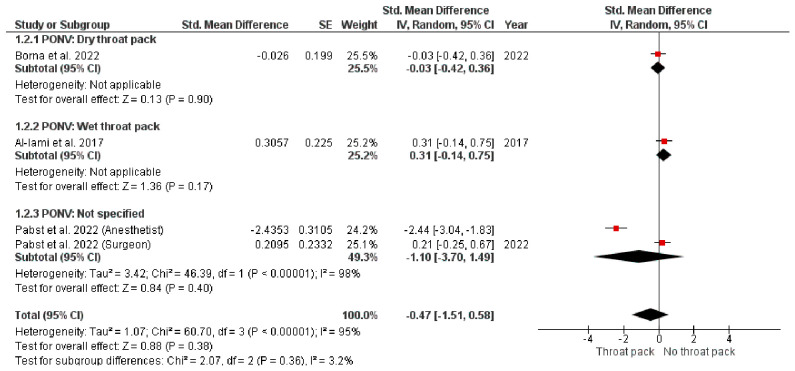
Forest plot of PONV [9,17,22].

Three studies were included in the meta-analysis for PONV and related symptoms. No statistically significant differences were observed between the throat pack and control groups for the overall population. The pooled effect was SMD = −0.47 (95% CI: −1.51 to 0.58). The prediction interval ranged from −4.11 to 3.17. Similarly, no significant results were found in any of the analyzed subgroups (Wet, Dry, or Not Specified). Figure 3 shows the forest plot for the PONV variable.

#### 3.5.3. Ultrasound Measurement

**Figure 4:**
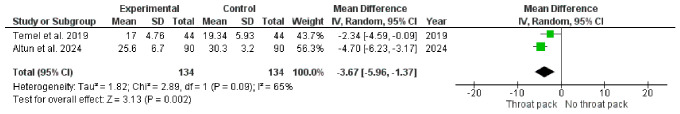
Forest plot for anteroposterior diameter (mm) ultrasound: throat pack versus no throat pack [14,16].

**Figure 5:**
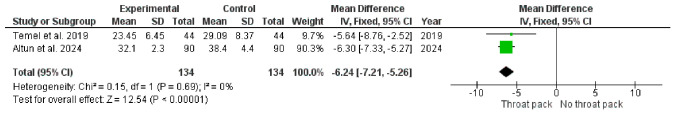
Forest plot for cranio-caudal diameter (mm) ultrasound: throat pack versus no throat pack [14,16].

**Figure 6:**
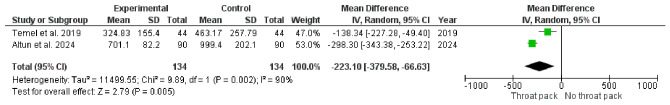
Forest plot for antral cross-sectional area (mm^2^) ultrasound: throat pack versus no throat pack [14,16].

Two studies provided data for three distinct meta-analyses: anteroposterior diameter (mm), cranio-caudal diameter (mm), and antral cross-sectional area (mm^2^). All three ultrasound parameters yielded statistically significant results: anteroposterior diameter (MD = −3.67; 95% CI [−5.96, −1.37]; *p* = 0.002; prediction interval −11.99 to 4.65), cranio-caudal diameter (MD = −6.24; 95% CI [−7.21, −5.26]; *p* < 0.00001; prediction interval −7.21 to −5.26), and antral cross-sectional area (MD = −223.10; 95% CI [−379.58, −66.63]; *p* = 0.005; prediction interval −955 to 509). The corresponding forest plots for these variables are displayed in Figure 4, Figure 5 and Figure 6.

#### 3.5.4. Assessment of Publication Bias and Sensitivity Analysis

Publication bias was not formally assessed for either pain intensity or PONV, as established methodological guidelines indicate that such analyses lack sufficient statistical power and are not considered reliable when fewer than 10 studies are included in a meta-analysis.

Regarding pain intensity, the sensitivity analysis showed no substantial changes in the overall effect size or statistical significance upon the systematic exclusion of individual studies, confirming the robustness of the results (Table S7). In contrast, the sensitivity analysis for the PONV meta-analysis indicated that the high initial heterogeneity (I^2^ > 90%) was primarily driven by a single study. Upon the exclusion of this outlier, the I^2^ value decreased to 0%, and the pooled effect size shifted in direction, with the SMD changing from positive to negative (Table S8). Despite this reversal in direction, the results remained statistically non-significant in both scenarios. These findings suggest that, while the initial inconsistency was attributable to one specific study, the overall conclusion regarding the lack of a significant difference between groups for PONV remains stable. Sensitivity analysis was not performed for the ultrasound measurement meta-analyses, as only two studies were included. The completed PRISMA checklist is provided as Table S9.

## 4. Discussion

The present investigation consists of a systematic review with meta-analysis that examines the application of pharyngeal packing in maxillofacial and upper airway surgeries. The analysis focuses on its potential to minimize the aspiration of fluids and other elements, its influence on the occurrence of Postoperative Nausea and Vomiting (PONV), and its effect on gastric volume. Likewise, the possible adverse effects related to its use, such as pharyngeal discomfort, are considered.

Prediction intervals were wide for most outcomes and often crossed the null value, suggesting that the effect in a future study may differ substantially from the pooled estimate, particularly in the presence of between-study heterogeneity.

The results, based exclusively on Randomized Controlled Trials, suggest that the routine practice of using a throat pack in maxillofacial and upper airway surgeries is not supported by sufficient evidence. The statistical analyses indicated that the intervention is associated with a significant increase in the severity of postoperative throat pain, an adverse effect that can manifest immediately and remains documented up to 24 h post-surgery. However, regarding Postoperative Nausea and Vomiting (PONV), no statistically significant differences were observed between the groups. Given the clinical heterogeneity, the limited number of studies, and the very low certainty of the evidence according to the GRADE assessment, current data suggest that the routine use of these packs does not decrease PONV incidence and may worsen the patient’s throat discomfort; therefore, these findings should be interpreted with caution.

Over the years, numerous bibliographic and systematic reviews have consistently demonstrated that throat packs are ineffective in preventing Postoperative Nausea and Vomiting (PONV) and, conversely, contribute to postoperative throat discomfort, findings that align with the results of the present study. However, previous research often faces significant methodological limitations. This underscores the necessity for a systematic review that implements a rigorous selection process, robust and reliable meta-analyses, and comprehensive data integration to finally reach a definitive conclusion on the matter.

The most recent publication on this matter is by Saha et al. [23] in 2025, who conducted a systematic review with meta-analysis that included the only two clinical trials in which orthognathic surgery was performed, and which have also been included in the present study [10,15]. Their article concluded that there is no significant difference in PONV and that the intervention causes throat pain, with both results assessed up to 2 h post-surgery. The present study has increased the number of trials implemented, finding that the use of a throat pack is associated with postoperative throat pain both immediately and up to 24 h after the procedure; furthermore, it extends the findings regarding PONV, demonstrating that the intervention lacks a significant preventive effect up to 4 h post-surgery. Previously, Casenave et al. [24] in 2023 published a systematic review that included seven articles implemented in the present study [12,13,15,16,17,18,19] and included two articles discarded for using anesthetic medication in the throat pack [25,26]. Their article showed that the throat pack is not effective in preventing PONV and causes throat discomfort; however, to obtain these results, the study combined different data collection points close in time, both in the immediate and later postoperative periods, without differentiating between specific hours. The present study improves upon this methodology by increasing the number of included studies, discarding trials that involve pharmacological treatment within the throat pack itself, and categorizing data collection points by specific time intervals. Another researcher named Xie et al. [27] in 2021 published a systematic review with meta-analysis presenting seven articles that coincide with the selection of the present study [12,13,15,16,17,19,22], and additionally, a pilot study [28], a study that includes a group with a benzodiazepine-soaked pack [26] and a study that includes children [29]. In their meta-analysis, it can be observed that different measurements from the same study were used to perform the analysis. No strategies were mentioned to prevent “dDouble cCounting,” which biases the precision of the results. Furthermore, the study does not report the specific results of homogeneity tests, only mentioning in the discussion that homogeneity is “relatively low”. The present study improves upon this research methodology with a more rigorous selection of studies and more robust, reliable statistical results. On the other hand, Anderson et al. [30] in 2020 published a systematic review on the throat pack that included 13 scientific articles with diverse methodologies; however, they only reported qualitative results without performing a meta-analysis. The present study has implemented a superior selection of studies and a robust statistical analysis through meta-analysis, which provides significantly greater strength and reliability to the obtained results. Furthermore, Jin et al. [31] published a systematic review on the throat pack in 2019 that included ten articles, five of which have been included in the present study [12,13,18,19,20]. Additionally, that study included two articles discarded from the present study for using medication within the throat pack itself [26,31], two articles involving different throat pack groups [32,33], and finally, one article discarded due to low quality [34]. Moreover, in their meta-analysis, they used different measurements from the same article to analyze the same statistic without mentioning strategies to avoid the “‘double counting”’ of subjects. The present study offers results derived from a more rigorous selection of studies and a more robust and reliable meta-analysis. And finally, Athanassoglou et al. [35] published a literature review in 2018 featuring a qualitative analysis of 45 articles. Their study employed a diverse methodology, encompassing clinical trials, literature reviews, opinion pieces, and anesthesia-related articles. The inclusion criteria were broad, requiring only that the articles be written in English and focus on the use of throat packs during anesthesia; the only exclusion criterion was the lack of access to the full-text version. This study, which involved a more rigorous search process, concludes that there is no evidence to support the routine use of throat packs. Furthermore, it reports significant complications associated with their use, ranging from intubation difficulties necessitating re-intubation to patient mortality.

The objectives of this investigation were satisfactorily met. It was possible to determine whether the presence of a pharyngeal pack affected the outcome variables of throat pain and PONV. Despite the fact that the results did not show the expected effect, the investigation provided valuable and well-founded data that contribute to the debate on the utility of this surgical practice. The methodological robustness of the design allowed for the obtaining of reliable conclusions regarding the lack of efficacy of the pack for the variables studied.

The main limitation identified lies in the high heterogeneity of the treatment protocols implemented in the primary studies included, as this variability encompasses everything from the specific surgical procedures performed to the post-surgical recommendations and the medication regimen administered to the subjects, which extends to a significant diversity in anesthesia techniques and postoperative care schemes that could subtly influence pain perception and the local inflammatory response. This methodological diversity, including the administration of different analgesic drugs and variations in recovery guidelines, acts as a potential confounding variable that limits the ability to generalize the exact magnitude of the observed effect despite the statistical consistency achieved, which underscores the complexity of synthesizing findings from clinical environments with such diverse perioperative management standards.

Additionally, the existence of incomplete or missing data was confirmed in numerous publications and the impossibility of correcting this lack of information despite attempts to contact the authors restricted the extraction of crucial data for a more exhaustive meta-analysis, thereby compromising the capacity for integral synthesis and the absolute robustness of the conclusions obtained in some specific outcomes due to this lack of standardization in variable reporting. This lack of detailed information regarding secondary outcomes and adverse events in some studies limits the global interpretation of the procedure’s safety, which reinforces the need for future research to follow stricter reporting protocols to facilitate precise comparisons between different surgical populations.

Furthermore, the variability in outcome measurement tools across the primary studies, such as the alternating use of the Visual Analog Scale (VAS) and the Numeric Pain Rating Scale (NPRS) for pain, as well as the Korttila scale for PONV, presents a challenge for data synthesis. This lack of uniformity in clinical variable reporting, combined with the heterogeneity in anesthesia protocols and postoperative care regimens previously described, limits the ability to isolate with total purity the impact of oropharyngeal packing from other concurrent perioperative interventions.

There is significant variability among secondary outcomes due to a lack of standardization in the variables reported. This inconsistency in data collection prevents the synthesis of results into a unified analysis.

A primary limitation of this meta-analysis lies in the restricted number of included studies, a circumstance that precluded the use of robust statistical tests to assess publication bias. This scarcity of evidence was particularly notable regarding ultrasound-related analyses; therefore, caution is warranted when interpreting these specific results. Furthermore, the robustness of the quantitative synthesis was compromised by the fact that several studies reported outcomes solely as percentages of subjects, rather than the means and standard deviations required for inclusion in the formal statistical analysis.

Finally, the sensitivity analysis revealed that one specific study acted as a significant source of distortion, disproportionately influencing the overall heterogeneity. This finding underscores the importance of considering individual methodological factors that may skew general trends and highlights the need for future research utilizing more standardized measurement protocols.

A study by Henderson et al. [36] published in 2025, based on the England National Health Service (NHS) database, warns about the risks of throat packs by identifying them as a significant cause of retained foreign objects in patients. After analyzing eleven years of records, the authors identified 40 incidents classified as “never events,” representing 3.2% of all cases involving accidentally retained surgical items.

The debate surrounding the clinical utility of throat packs has been analyzed from the perspective of professional practice. According to one study, 75% of the surveyed specialists choose to use them, perceiving the procedure as safe. However, the study also notes that 26.1% of professionals reported being aware of at least one incident in their unit, reflecting a variability between general safety perceptions and the frequency of events recorded in clinical settings [37].

The cross-sectional study conducted among 243 anesthesiologists revealed that, while the use of oropharyngeal throat packs is highly prevalent (favored by 75.3% for oral surgeries) and typically managed by anesthesiologists, there is an incidence of safety complications. The most critical finding was that 38.27% of participants reported instances of retained (forgotten) packs, primarily attributed to team changes and communication failures, which led to postoperative airway obstruction in 37.6% of those cases. Although individual preventive measures are employed, such as leaving a portion of the pack outside the mouth (63.4%), less than half of the respondents (42.8%) followed a standardized institutional protocol, underscoring the urgent need for safety guidelines, visual verification, and strict documentation in surgical checklists [38].

An investigation of postoperative stomach contents, performed by aspiration, has demonstrated identical results with or without the use of a pharyngeal pack, suggesting no difference in outcome regarding the presence of blood in the stomach contents [8].

The use of a throat pack in maxillofacial surgery is warranted as an additional protective barrier in a high-risk context, yet its value must be weighed against its adverse effects. It is essential to recognize that the inflated cuff of the endotracheal tube already constitutes the primary mechanism for airway protection, as it is indispensable for the tracheal seal, ensuring positive-pressure mechanical ventilation, and preventing the passage of fluids. Therefore, the pack’s use complements this essential protection by intercepting blood and surgical debris at the pharyngeal level before they can reach the cuff, a risk inherent to this type of surgery.

Finally, the widespread use of medications that directly influence primary outcomes serves as a significant confounding factor across the analyzed studies. Specifically, six articles reported the administration of antiemetic drugs, either as standard prophylaxis or rescue therapy [9,10,13,15,17,19]. Furthermore, nearly all included studies [9,10,11,12,13,14,15,16,17,18,19] utilized analgesics for routine or rescue pain management. This pharmacological intervention represents a critical limitation, as it likely masks the actual clinical effect of the throat pack on nausea and pain. However, it must be noted that withholding such medication to achieve a pure observation would present substantial ethical challenges.

Based on our findings, it is recommended that the routine practice of placing pharyngeal packs in surgeries be reconsidered, given that it does not appear to offer a significant benefit in the reduction in PONV and throat pain. For future research, we suggest the inclusion of a larger sample size to increase statistical power and be able to detect more subtle differences. It would be valuable to explore other methods for the prevention of PONV in this type of surgery, as well as to investigate the impact of the pack on other variables.

## 5. Conclusions

In conclusion, current evidence does not demonstrate a clear benefit for the routine use of oropharyngeal packing, and suggests a possible increase in postoperative throat discomfort. While acknowledging the limited number of studies, significant clinical heterogeneity, and the influence of perioperative co-interventions, these findings should be interpreted with caution. At present, there is insufficient high-quality evidence to support the routine use of pharyngeal packs for airway protection in maxillofacial and upper airway procedures. Further large-scale, well-controlled randomized trials are necessary to provide more definitive clinical recommendations.

## Figures and Tables

**Table 1:** Summary of findings and certainty of the evidence according to the GRADE approach.

Certainty Assessment							N. of Patients		Effect		Certainly	Impo.
N. Studies	Study Design	Risk of Bias	Inc.	Indirectly Evidence	Imp.	Others	Int	Comp	Rel	Abs		
Pain 6	RTs	S ^a^	S ^b^	Not serious	S ^c^	None	228	301	-	SMD 0.35 (0.09 to 0.62)	⨁◯◯◯ VERY LOW	NOT
PONV 3	RTs	S ^a^	S ^b^	Not serious	S ^c^	None	128	164	-	SMD −0.47 (−1.51 to 0.58)	⨁◯◯◯ VERY LOW	NOT
Figure 4 2	RTs	S ^a^	S ^b^	Not serious	S ^c^	None	134	134	-	MD −3.67 (−5.96 to −1.37)	⨁◯◯◯ VERY LOW	NOT
Figure 5 2	RTs	S ^a^	S ^b^	Not serious	S ^c^	None	134	134	-	MD −6.24 (−7.21 to −5.25)	⨁◯◯◯ VERY LOW	NOT
Figure 6 2	RTs	S ^a^	S ^b^	Not serious	S ^c^	None	134	134	-	MD −223.1 (−379.58 to −66.63)	⨁◯◯◯ VERY LOW	NOT

PONV: Postoperative Nausea and Vomiting; RTs: randomized trials; SMD: Standardized Mean Difference; MD: Mean Difference; S: serious; Int: intervention; Comp: comparison; Rel: relative (95% CI); Abs: absolute (95% CI); Inc: inconsistency; Imp: imprecision; Impo.: importance; ^a^. this variability may be due to the fact that the studies present differences in the interventions; ^b^. these differences may be because the studies present differences in the interventions; ^c^. the results show differences in the confidence interval. Quality of evidence according to the GRADE approach: ⨁◯◯◯ Very low.

## Data Availability

The raw data supporting the conclusions of this article will be made available by the authors on request.

## Supplementary Materials

The following supporting information can be downloaded at: https://www.mdpi.com/article/10.3390/dj14060337/s1, Table S1: Search strategy for each database; Table S2: Main characteristics of included studies using dry throat packs; Table S3: Main characteristics of included studies using wet throat packs; Table S4: Demographic and clinical baseline of participants (Age, Gender, BMI); Figure S1: Methodological quality assessment using the Cochrane Risk of Bias tool (RoB 2); Table S5: Intraoperative variables (Anesthesia and surgery duration, blood loss volume); Table S6: Postoperative long-term variables (Sore throat, PONV, and Gastric volume ultrasound); Table S7. Sensitivity analyses based on various exclusion criteria for pain intensity; Table S8. Sensitivity analyses based on various exclusion criteria for Postoperative Nausea and Vomiting (PONV) and related symptoms. Table S9: PRISMA 2020 Checklist.

## Abbreviations

The following abbreviations are used in this manuscript:

PONV	Postoperative Nausea and Vomiting
WOS	Web of Science
MeSHs	Medical Subject Headings
RCTs	Randomized Controlled Trials
Rob2	Cochrane Risk of Bias
SMD	Standardized Mean Difference
OR	Odds Ratio
FE	Fixed-Effects Model
CI	Confidence Interval
K	Number of Studies
I^2^	Heterogeneity
P-val	Statistical Significance
PRISMA	Preferred Reporting Items for Systematic Reviews and Meta-Analyses
VAS	Visual Analogic Scale
NPRS	Numeric Pain Rating Scale
GRADE	Grading of Recommendations, Assessment, Development and Evaluation
SD	Standard Deviation
Q	Cochrane Q Heterogeneity
