# Supplementary material for: Impact of Oropharyngeal Packing on Postoperative Nausea, Vomiting, and Throat Discomfort: A Systematic Review and Meta-Analysis
Source: Dent J (Basel). 2026 Jun 2;14(6):337. doi: 10.3390/dj14060337 (PMC13297648; doi:10.3390/dj14060337)
Supplement: Supplementary file 1 [file dentistry-14-00337-s001.zip › dentistry-4016770-supplementary.pdf]

Supplementary Material

Table S1. Search strategy by database

Database	Search strategy
Pubmed	(("Surgical Sponges"[MeSH Terms] OR "sponge*"[Title/Abstract] OR "throat swab*"[Title/Abstract] OR "throat pack*"[Title/Abstract] OR "tampons, surgical"[MeSH Terms] OR "tampon*"[Title/Abstract]) AND ("surgery, oral"[MeSH Terms] OR "exodontics"[Title/Abstract] OR "Oral Surgical Procedures"[MeSH Terms] OR "procedure*"[Title/Abstract] OR "Orthognathic Surgical Procedures"[MeSH Terms] OR "surger*"[Title/Abstract] OR "Orthognathic Surgery"[MeSH Terms] OR "Mandibular Reconstruction"[MeSH Terms] OR "reconstructi*"[Title/Abstract] OR "Anesthesia"[MeSH Terms] OR "Anesthesia"[Title/Abstract] OR "anesthesia, general"[MeSH Terms] OR "General"[Title/Abstract] OR "anesthesia, endotracheal"[MeSH Terms] OR "Intratracheal"[Title/Abstract] OR "Endotracheal"[Title/Abstract])) AND ((clinicaltrial[Filter] OR randomizedcontrolledtrial[Filter]) AND (alladult[Filter]))
Scopus	(TITLE-ABS-KEY("Surgical Sponges" OR sponge* OR "throat swab*" OR "throat pack*" OR "Tampons, Surgical" OR tampon*)) AND (TITLE-ABS-KEY("Surgery, Oral" OR exodontics OR "Oral Surgical Procedures" OR procedure* OR "Orthognathic Surgical Procedures" OR surger* OR "Orthognathic Surgery" OR "Mandibular Reconstruction" OR reconstructi* OR Anesthesia OR "Anesthesia, General" OR General OR "Anesthesia, Endotracheal" OR Intratracheal OR Endotracheal))
Web of Science	(("Surgical Sponges" OR "sponge" OR "throat swab" OR "throat pack" OR "Tampons, Surgical" OR "tampon") AND ("Surgery, Oral" OR exodontics OR "Oral Surgical Procedures" OR procedure* OR "Orthognathic Surgical Procedures" OR "surger" OR "Orthognathic Surgery" OR "Mandibular Reconstruction" OR "reconstructi") AND (Anesthesia OR "Anesthesia, General" OR General OR "Anesthesia, Endotracheal" OR Intratracheal OR Endotracheal)) (Topic) AND Clinical Trial (Document Types)
Cochrane Library	(("Surgical Sponges" OR "sponge" OR "throat swab" OR "throat pack" OR "Tampons, Surgical" OR "tampon") AND ("Surgery, Oral" OR exodontics OR "Oral Surgical Procedures" OR procedure* OR "Orthognathic Surgical Procedures" OR "surger" OR "Orthognathic Surgery" OR "Mandibular Reconstruction" OR "reconstructi") AND (Anesthesia OR "Anesthesia, General" OR General OR "Anesthesia, Endotracheal" OR Intratracheal OR Endotracheal)) AND trials

Table S2. Characteristics of studies using dry throat packs

Authors	Surgery	Post-surgery
Borna et al., 2022 [9]	Septorhinoplasty	Dexamethasone 10 mg (after induction), Ondansetron 4 mg (30 min before procedure completion), Acetaminophen 1g IV (after incision), Fentanyl 50-200 mcg, Hydromorphone 0.2-0.8 mg (as rescue medications for pain control)
Powel et al., 2022 [10]	Orthognathic surgery	Scopolamine extended-release transdermal film (1.5 mg), oral acetaminophen (1000 mg), intravenous dexamethasone (8 mg). Intraoperatively: intravenous ondansetron (4 mg) 30-60 minutes before surgery end. Postoperatively: non-opioid pain medications and rescue medications for PONV as needed.
Al-Jandan et al., 2018 [11]	Elective nasal surgery (septoplasty, septo-rhinoplasty and FESS)	Amoxicillin (500 mg every 8h), ibuprofen (400 mg every 8h), and chlorhexidine (0.2% MW qid)
Green et al., 2017 [12]	FESS	Patients were routinely given oxycodone 5 mg/acetan acetaminophen 325 mg every 6 hours as needed for pain, but how much of the medication they took is unclear because we did not study this.
Korkut AY et al., 2010 [13]	Routine nasal surgery (septoplasty, septorhinoplasty, ESS, ESS with septoplasty)	Tenoxicam 20 mg IV, neostigmine 1.5 mg and atropine 0.5 mg (for reversal of neuromuscular blockade). Metoclopramide 10 mg (for moderate/severe nausea or 2+ emetic episodes).

FESS: functional endoscopic sinus surgery; mg: milligram; kg: Killogram; μg: microgram; h: hours; MW: mouth wash; qid: quater in die; min: minute; ESS: endoscopic sinus surgery.

Table S3. Characteristics of studies using wet throat packs

Authors	Surgery	Post-surgery
Altun et al., 2024 [14]	FESS, septoplasty and septorhinoplasty	Atropine 0.5 mg and neostigmine 1.5 mg (to reverse neuromuscular blockade). Paracetamol for severe sore throat.
Faro et al., 2020 [15]	Orthognathic surgery (sagittal split osteotomy, Le Fort I osteotomy, chin osteotomy)	Dexamethasone (10 mg), parecoxib sodium (40 mg), dipyrone (3-4 g), magnesium sulphate (40 mg/kg), ketamine (0.25 mg/kg), pantoprazole (40 mg), ondansetron (8 mg), dimenhydrinate (30 mg) intraoperatively. Morphine (0.1 mg/kg) IV before end of surgery. Sugammadex (2-4 mg/kg) for reversal. Post-op: dipyrone 2g q6h, parecoxib sodium 40mg daily, pantoprazole 40mg daily. Rescue: ondansetron 4mg q8h (nausea/vomiting), tramadol 50mg + ondansetron 4mg q8h (pain).
Temel et al., 2019 [16]	Elective nasal surgery (septoplasty, septo-rhinoplasty and FESS)	Ranitidine (0.5 mg/kg) IV (after intubation), tenoxicam (20 mg) and tramadol (1 mg/kg) (at end of operation). Atropine 0.5 mg and neostigmine 1.5 mg (for reversal).
Al-lami et al., 2017 [17]	FESS, septonhinoplasty, septoplasty, reduction of inferior turbinates, nasal polypectomy and divisions of nasal adhesions.	Paracetamol IV and NSAIDs if no contraindication. Standardized anti-emetic regimen. Nasal tampons for 4 hours post-op in both groups for more blood losing operations.
Razavi et al., 2015 [18]	Septorhinoplasty, nasal surgery, head and neck surgery, sinus surgery.	In the moderate pain group, a non-opioid sedative was administered and in the severe pain group intramuscular morphine was used after surgery.
Piltcher et al., 2007 [19]	Nasal and/or paranasal sinus surgeries	Paracetamol and dipyrone (postoperative analgesia). Opioids avoided. Dexamethasone 0.05 mg/kg, Tenoxicam 40 mg. Prophylactic metoclopramide or ondansetron were not used.
Basha et al., 2006 [20]	Septoplasty, Turbinectomy and Inferior Turbinectomy, Septoplasty + Turbinectomy and Inferior Turbinectomy, Functional Endoscopic Sinus Surgery, Polypectomy, Rhinoplasty, Septorhinoplasty, Submucous diathermy to turbinates.	At the end of surgery, neostigmine 2.5 mg and glycopyrrolate 500 μg were administered to reverse neuromuscular blockade.
Tay et al., 2002 [21]	Minor oral surgery (impacted wisdom teeth removal)	Neostigmine 2.5 mg and atropine 0.6 mg (to reverse neuromuscular blockade)
Pabst et al., 2022 [22]	upper airway surgical intervention	

FESS: functional endoscopic sinus surgery; NSAID: Non-Steroidal Anti-Inflammatory Drug; mg: milligram; kg: Kilogram; μg: microgram.

Table S4. Participant demographics and clinical baseline

Intervention	Authors	Group	Age Mean ± SD		Gender Male(n)/ Female(n)		BMI mean ± SD	
			Int	Cont	Int	Cont	Int	Cont
Dry	Borna et al., 2022 [9]	Int: 51 Cont: 50	35±12.1	30.3±9.4	10/40	11/40	-	-
	Powel et al. 2022 [10]	Int: 15 Cont: 15	32.13 ± 12.98	26.2 ± 13.27	6/9	6/9	-	-
	Al-Jandan et al.,2018 [11]	Int: 40 Cont: 40	27.3		45/35		-	-
	Green et al., 2017 [12]	Int: 23 Cont: 23	51.8±17	49.9±15	15/8	14/8	26.8 ± 4.3	28.7±6.2
	Korkut AY et al., 2010 [13]	Int: 50 Cont: 50	31.5±11.95	27.98±13.14	29/21	22/28	24.7±4.28	25.84±3.57
Wet	Altun et al., 2024 [14]	Int: 90 Cont: 90	39.2 ± 11	38.3 ± 7.8	47/43	46/44	25.2±2.7	25.1±3.9
	Faro et al., 2020 [15]	Int: 27 Cont: 27	29.44±8.53		19/36		-	-
	Temel et al., 2019 [16]	Int: 44 Cont: 44	30.18 ± 8.98	32.36 ± 10.2	19/15	27/17	24.28±4.13	25.57±4.5
	Al-lami et al., 2017 [17]	Int: 40 Cont: 40	42	44	24/16	33/7	-	-
	Razavi et al., 2015 [18]	Int: 44 Cont: 45	27.02 ± 7.08	27.18 ± 7.08	5/39	5/40	-	-
	Piltcher et al., 2007 [19]	Int: 70 Cont: 74	35.4	34.6	46/24	45/29	-	-
	Basha et al., 2006 [20]	Int: 45 Cont: 48	34	34	35/10	34/14	-	-
	Tay et al., 2002 [21]	Int: 26 Cont: 36	-	-	-	-	-	-
Undefined	Pabst et al., 2022 [22]	Int: 74 Cont: 74	40 ± 21	53 ± 22	39/29	34/34		

Int: intervention; Cont: control; SD: Statistical deviation; BMI: Body mass index.

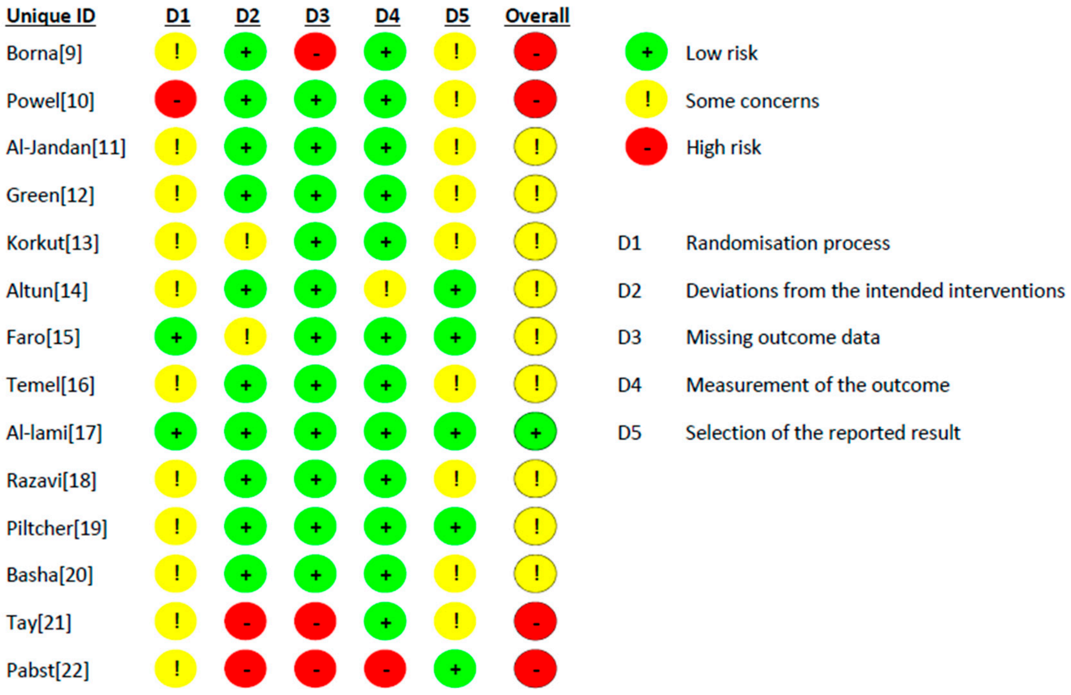

Figure S1. Risk of bias assessment (RoB 2)

Table S5. Intraoperative data: Anesthesia and Surgery duration

Intraoperative variable	Intervention	Study	Outcome	
			Throat pack	Control
Anesthesia duration	Dry	Borna et al., [9]<br>Mean ± SD; minutes	225.9 ± 62.4	249.9 ± 71.6
		Green et al., [12]<br>Mean ± SD; minutes	210 ± 72	216 ± 84
		Razavi et al., [18]<br>Mean ± SD; minutes	169.09 ± 29.71	172.89 ± 37.45
Surgery duration	Dry	Borna et al., [9]<br>mean ± SD; minutes	192.6 ± 70.5	209.9 ± 69.5
		Al-Jandan et al., [11]<br>mean ± SD; minutes	75 ± 25	
		Green et al., [12]<br>mean ± SD; minutes	144 ± 66	156 ± 96
	Wet	Altun et al., [14]<br>mean ± SD; minutes	157.8 ± 47.8	161 ± 45.9
		Faro et al., [15]<br>mean ± SD; minutes	259.28 ± 70.5	253.64 ± 70.93
		Temel et al., 2019 [16]<br>mean ± SD; minutes	259.28 ± 70.5	253.64 ± 70.93
		Tay HYY et al., [21]	No statistical difference	
Blood loss volume	Dry	Green et al., [12]<br>mean ± SD: ml	77 ± 76	72 ± 75
	Wet	Altun et al., [14]<br>mean ± SD: ml	165.7 ± 32.7	106.8 ± 32.7
		Faro et al., [15]<br>mean ± SD; ml	588 ± 312.42	
Bloody	Dry	Powell et al., [10]<br>n (%)	10(66.7)	10(66.7)
Difficulty of swallowing	Dry	Borna et al., [9]<br>mean ± SD	Post day = 2.56±1.51<br>24h = 2.73.±1.37<br>48h = 2.42±1.57	Post day = 3.06±1.32<br>24h = 2.28±1.62<br>48h = 1.92±1.37

Swelling in throat	Dry	Borna et al., [9]<br>mean ± SD	Post day = 2.40±1.36<br>24h = 2.90±1.49<br>48h = 2.36±1.65	Post day = 3.12±1.6<br>24h = 2.18±1.56<br>48h = 2.94±1.35

SD: Standard deviation; VAS: Visual analog scale; Pre: preoperative; Post: postoperative; FESS: functional endoscopic sinus surgery; bloody: presence of bloody gastric content.

Table S6. Long-term postoperative outcomes (Sore throat, PONV)

Postoperative variables	Intervention	Study	Outcome	
			Control	Throat pack
Sore throat	Dry	Borna et al., [9]<br>mean ± SD	Recovery = 2.78±1.56<br>24h = 2.48±1.55<br>48h = 2.10±1.39	Recovery = 3.65±1.47<br>24h = 3.18±1.51<br>48h = 3.25±1.62
		Powell et al., [10]<br>n (%)<br>mean ± SD	2h = 7 (46.7)<br>2h = 2.8 ± 3.5	2h = 12 (80)<br>2h = 4.1 ± 2.4
		Al-Jandan et al., [11]	n (%) = 20(50)	n (%) = 37(92.5)
		Green et al., [12]<br>mean ± SD	4h = 1.82±2.5<br>24h = 1.83±2.5	4h = 1.65±2.4<br>24h = 0.3±1.3
	Wet	Altun et al., [14]<br>n (%)	Recovery = 32 (35.6)<br>Ward = 24 (26.7)	Recovery = 32 (35.6)<br>Ward = 2 (2.2)
		Faro et al., [15]<br>mean ± SD	2h = 2.72 ± 3.91<br>24h = 0.97 ± 2.27	2h = 4.76 ± 3.71<br>24h = 3.52 ± 3.25
		Al-lami et al., [17]<br>mean ± SD	Recovery = 1.3 ± 2.5<br>2h = 2.3 ± 2.8<br>6h = 1.6 ± 2.4	Recovery = 2.5 ± 2.8<br>2h = 2.1 ± 2.4<br>6h = 1.4 ± 1.6
		Razavi et al., [18]<br>n (%)	Recovery = 12 (27)<br>2h = 8 (18)<br>6h = 5 (11)<br>24h = 10 (22)	Recovery = 17 (39)<br>2h = 14 (32)<br>6h = 7 (16)<br>24h = 14 (32)
		Basha et al., [20]<br>n (%)	Recovery = 30 (66)	Recovery = 16 (33)
		Tay HYY et al., [21]<br>n (%)	Recovery = 28 (78)<br>24h = 20 (56)	Recovery = 19 (73)<br>24h = 13 (50)
PONV	Dry	Borna et al., [9]<br>mean ± SD	Recovery = 0.60±1.39<br>24h = 0.42±1.28<br>48h = 0.2±0.61	Recovery = 0.55±1.08<br>24h = 0.39±1.00<br>48h = 0.04±0.20
		Powell et al., [10]<br>n (%)	2h = 3 (20)<br>24h = 4 (26.7)	2h = 3 (20)<br>24h = 4 (26.7)
		Al-Jandan et al., [11]<br>n (%)	4h = 0 (0)	4h = 4 (10)
		Green et al., [12]<br>n (%)	4h = 1(4.3)<br>24h = 1(4.3)	4h = 5(21.7)<br>24h = 2(8.7)
	Wet	Altun et al., [14]<br>n (%)	Recovery = 42 (46.7)<br>2h = 49 (54.4)	Recovery = 23 (25.6)<br>2h = 5 (5.6)

		Faro et al., [15]<br>n (%)	24h = 9 (36.0)	24h = 9 (36.0)
		Temel et al., [16]<br>%	Septoplasty<br>2h = 60<br>4h = 60<br>8h = 53.3<br>24h = 13.3<br><br>Septorhinoplasty<br>2h = 50<br>4h = 41.7<br>8h = 33.3<br>24h = 16.7<br><br>FESS<br>2h = 54.5<br>4h = 47.7<br>8h = 38.6<br>24h = 15.9	Septoplasty<br>2h = 37.5<br>4h = 18.8<br>8h = 50<br>24h = 0<br><br>Septorhinoplasty<br>2h = 50<br>4h = 40<br>8h = 25<br>24h = 5<br><br>FESS<br>2h = 40.9<br>4h = 29.5<br>8h = 31.8<br>24h = 2.3
		Al-lami et al., [17]<br>mean ± SD	6h = 0.36 ± 1.39	6h = 2.75 ± 10.86
		Basha et al., [20]<br>n (%)	Recovery = 7 (15)	Recovery = 15 (33)
		Tay HYY et al., [21]<br>Mean	0	0
Gastric volume<br>(Ultrasound)	Wet	Altun et al., [14]<br>mean ± SD	Anteroposterior diameter (mm)<br>Pre = 23.6 ± 2<br>Post = 30.3 ± 3.2<br><br>Cranio-caudal diameter (mm)<br>Pre = 29.7 ± 2.5<br>Post = 38.4 ± 4.4<br><br>Antral cross-sectional area (mm2)<br>Pre = 599.1 ± 93<br>Post = 999.4 ± 202.1<br><br>Gastric volume (ml)<br>Pre = 87.2 ± 13.5<br>Post = 134.5 ± 27.2	Anteroposterior diameter (mm)<br>Pre = 23.6 ± 1.5<br>Post = 25.6 ± 6.7<br><br>Cranio-caudal diameter (mm)<br>Pre = 29.6 ± 2.4<br>Post = 32.1 ± 2.3<br><br>Antral cross-sectional area (mm2)<br>Pre = 594.1 ± 71.5<br>Post = 701.1 ± 82.2<br><br>Gastric volume (ml)<br>Pre = 86.5 ± 10.3<br>Post = 94.3 ± 11
		Temel et al., [16]<br>mean ± SD	Anteroposterior diameter (mm)<br>Pre = 14.82 ± 3.36<br>Post = 19.34 ± 5.93	Anteroposterior diameter (mm)<br>Pre = 15.25 ± 3.72<br>Post = 17 ± 4.76

	Cranio-caudal diameter (mm)<br>Pre = 24.05 ± 4.87<br>Post = 29.09 ± 8.37	Cranio-caudal diameter (mm)<br>Pre = 21.66 ± 5.31<br>Post = 23.45 ± 6.45
	Antral cross-sectional area (mm2)<br>Pre = 280.06 ± 87.16<br>Post = 463.17 ± 257.79	Antral cross-sectional area (mm2)<br>Pre = 266.16 ± 109.25<br>Post = 324.83 ± 155.54

PONV: postoperative nausea and vomiting; SD: Standard deviation; Surgeon: throat pack placed by anesthesist: Throat pack collocated by anesthetic; Pre : Preoperative; Post: Postoperative; FESS: functional endoscopic sinus surgery

Table S7. Sensitivity analyses based on various exclusion criteria for pain intensity.

Excluded trials	No. Trials	SMD (95% CI)	*P* FOR SMD	I^2	*P* for heterogeneity
Borna et al., 2022[9]	5	0.31 (0.00-0.62)	0.05	52%	0.06
Powell et al., 2022[10]	5	0.35 (0.05-0.65)	0.02	58%	0.04
Green et al., 2017[12]	5	0.39 (0.10-0.69)	0.009	54%	0.05
Faro et al., 2019[15]	5	0.32 (0.02-0.62)	0.04	56%	0.05
Al-lami et al., 2017[17]	5	0.44 (0.19-0.70)	0.0007	32%	0.19
Pabst et al., 2022[22] (Surgeon)	5	0.40 (0.10-0.70)	0.009	52%	0.07
Pabst et al., 2022[22] (Anesthetist)	5	0.26 (0.02-0.50)	0.03	27%	0.23

SMD: standardized mean difference.

Table S8. Sensitivity analyses based on various exclusion criteria for Postoperative Nausea and Vomiting (PONV) and related symptoms.

Excluded trials	No. Trials	SMD (95% CI)	*P* FOR SMD	I^2	*P* for heterogeneity
Borna et al., 2022[9]	2	-0.63 (-2.19-0.93)	0.43	97%	<0.00001
Al-lami et al., 2017[17]	2	-0.73 (-2.15-0.69)	0.31	96%	<0.00001
Pabst et al., 2022[22] (Anesthetist)	2	0.15 (-0.10-0.39	0.25	0%	0.52
Pabst et al., 2022[22] (Surgenon)	2	-0.70 (-2.14-0.74)	0.34	96%	<0.00001

SMD: standardized mean difference.

Table S9. PRISMA 2020 Checklist

Section and Topic	Item #	Checklist item	Location where item is reported
**TITLE**			
Title	1	Identify the report as a systematic review.	PAGE 1
**ABSTRACT**			
Abstract	2	See the PRISMA 2020 for Abstracts checklist.	PAGE 1
**INTRODUCTION**			
Rationale	3	Describe the rationale for the review in the context of existing knowledge.	PAGE 2
Objectives	4	Provide an explicit statement of the objective(s) or question(s) the review addresses.	PAGE 2
**METHODS**			
Eligibility criteria	5	Specify the inclusion and exclusion criteria for the review and how studies were grouped for the syntheses.	PAGE 3
Information sources	6	Specify all databases, registers, websites, organisations, reference lists and other sources searched or consulted to identify studies. Specify the date when each source was last searched or consulted.	PAGE 2
Search strategy	7	Present the full search strategies for all databases, registers and websites, including any filters and limits used.	PAGE 3
Selection process	8	Specify the methods used to decide whether a study met the inclusion criteria of the review, including how many reviewers screened each record and each report retrieved, whether they worked independently, and if applicable, details of automation tools used in the process.	PAGE 3
Data collection process	9	Specify the methods used to collect data from reports, including how many reviewers collected data from each report, whether they worked independently, any processes for obtaining or confirming data from study investigators, and if applicable, details of automation tools used in the process.	PAGE 4
Data items	10a	List and define all outcomes for which data were sought. Specify whether all results that were compatible with each outcome domain in each study were sought (e.g. for all measures, time points, analyses), and if not, the methods used to decide which results to collect.	PAGE 4
	10b	List and define all other variables for which data were sought (e.g. participant and intervention characteristics, funding sources). Describe any assumptions made about any missing or unclear information.	PAGE 4
Study risk of bias assessment	11	Specify the methods used to assess risk of bias in the included studies, including details of the tool(s) used, how many reviewers assessed each study and whether they worked independently, and if applicable, details of automation tools used in the process.	PAGE 4
Effect measures	12	Specify for each outcome the effect measure(s) (e.g. risk ratio, mean difference) used in the synthesis or presentation of results.	PAGE 4
Synthesis methods	13a	Describe the processes used to decide which studies were eligible for each synthesis (e.g. tabulating the study intervention characteristics and comparing against the planned groups for each synthesis (item #5)).	PAGE 5
	13b	Describe any methods required to prepare the data for presentation or synthesis, such as handling of missing summary statistics, or data conversions.	PAGE 5
	13c	Describe any methods used to tabulate or visually display results of individual studies and syntheses.	PAGE 5
	13d	Describe any methods used to synthesize results and provide a rationale for the choice(s). If meta-analysis was performed, describe the model(s), method(s) to identify the presence and extent of statistical heterogeneity, and software package(s) used.	PAGE 5

Section and Topic	Item #	Checklist item	Location where item is reported
	13e	Describe any methods used to explore possible causes of heterogeneity among study results (e.g. subgroup analysis, meta-regression).	PAGE 5
	13f	Describe any sensitivity analyses conducted to assess robustness of the synthesized results.	PAGE 5
Reporting bias assessment	14	Describe any methods used to assess risk of bias due to missing results in a synthesis (arising from reporting biases).	PAGE 5
Certainty assessment	15	Describe any methods used to assess certainty (or confidence) in the body of evidence for an outcome.	PAGE 5
**RESULTS**			
Study selection	16a	Describe the results of the search and selection process, from the number of records identified in the search to the number of studies included in the review, ideally using a flow diagram.	PAGE 6
	16b	Cite studies that might appear to meet the inclusion criteria, but which were excluded, and explain why they were excluded.	PAGE
Study characteristics	17	Cite each included study and present its characteristics.	PAGE 6
Risk of bias in studies	18	Present assessments of risk of bias for each included study.	PAGE 11
Results of individual studies	19	For all outcomes, present, for each study: (a) summary statistics for each group (where appropriate) and (b) an effect estimate and its precision (e.g. confidence/credible interval), ideally using structured tables or plots.	PAGE 11
Results of syntheses	20a	For each synthesis, briefly summarise the characteristics and risk of bias among contributing studies.	PAGE 11
	20b	Present results of all statistical syntheses conducted. If meta-analysis was done, present for each the summary estimate and its precision (e.g. confidence/credible interval) and measures of statistical heterogeneity. If comparing groups, describe the direction of the effect.	PAGE 11
	20c	Present results of all investigations of possible causes of heterogeneity among study results.	PAGE 17
	20d	Present results of all sensitivity analyses conducted to assess the robustness of the synthesized results.	PAGE 15
Reporting biases	21	Present assessments of risk of bias due to missing results (arising from reporting biases) for each synthesis assessed.	PAGE 11
Certainty of evidence	22	Present assessments of certainty (or confidence) in the body of evidence for each outcome assessed.	PAGE 11
**DISCUSSION**			
Discussion	23a	Provide a general interpretation of the results in the context of other evidence.	PAGE 17
	23b	Discuss any limitations of the evidence included in the review.	PAGE 17
	23c	Discuss any limitations of the review processes used.	PAGE 17
	23d	Discuss implications of the results for practice, policy, and future research.	PAGE 18
**OTHER INFORMATION**			
Registration and protocol	24a	Provide registration information for the review, including register name and registration number, or state that the review was not registered.	PAGE 2
	24b	Indicate where the review protocol can be accessed, or state that a protocol was not prepared.	PAGE 2

Section and Topic	Item #	Checklist item	Location where item is reported
	24c	Describe and explain any amendments to information provided at registration or in the protocol.	PAGE 2
Support	25	Describe sources of financial or non-financial support for the review, and the role of the funders or sponsors in the review.	PAGE 19
Competing interests	26	Declare any competing interests of review authors.	PAGE 19
Availability of data, code and other materials	27	Report which of the following are publicly available and where they can be found: template data collection forms; data extracted from included studies; data used for all analyses; analytic code; any other materials used in the review.	

From: Page MJ, McKenzie JE, Bossuyt PM, Boutron I, Hoffmann TC, Mulrow CD, et al. The PRISMA 2020 statement: an updated guideline for reporting systematic reviews. BMJ 2021;372:n71. doi: 10.1136/bmj.n71.

This work is licensed under CC BY 4.0. To view a copy of this license, visit https://creativecommons.org/licenses/by/4.0/
